# Assessing the accuracy of the GPT-4 model in multidisciplinary tumor board decision prediction

**DOI:** 10.1007/s12094-025-03905-1

**Published:** 2025-03-25

**Authors:** Efe Cem Erdat, Merih Yalçıner, Mehmet Berk Örüncü, Yüksel Ürün, Filiz Çay Şenler

**Affiliations:** 1 Department of Medical Oncology, Ankara University Faculty of Medicine, Tip Fakultesi Cd. No 1 Mamak, Ankara, Turkey; 2 Cancer Institute, Ankara University, Ankara, Turkey

**Keywords:** Artificial intelligence, Tumor board, Cancer treatment, Multidisciplinary approach, Machine learning, Real-world data

## Abstract

**Purpose:**

Artificial intelligence models like GPT-4 (OpenAI) have the potential to support clinical decision-making in oncology. This study aimed to assess the consistency between multidisciplinary tumor board (MTB) decisions and GPT-4 model predictions in cancer patient management.

**Patients and methods:**

A cross-sectional study was conducted involving patients aged ≥ 18 years with definite or suspicious cancer diagnoses presented at MTBs in Ankara University Hospitals, Türkiye, from February 2021 to June 2023. GPT-4 was utilized to generate treatment recommendations based on case summaries. Three independent raters evaluated the compatibility between MTB decisions and GPT-4 predictions using a 4-point Likert scale. Cases with mean compatibility scores equal to or below 2 were reviewed by two expert oncologists for appropriateness.

**Results:**

A total of 610 patients were included. The mean compatibility score across raters was 3.59 (SD = 0.81), indicating high agreement between GPT-4 predictions and MTB decisions. Cronbach’s alpha was 0.950 (95% CI 0.935–0.960), demonstrating excellent interrater reliability. Sixty-two cases (10.2%) had mean compatibility scores below the threshold of 2. The first expert oncologist deemed GPT-4's predictions inappropriate in 8 of these cases (12.9%), while the second deemed them inappropriate in 16 cases (25.8%). Cohen’s kappa showed moderate agreement (*κ* = 0.50, 95% CI 0.25–0.75, *p* < 0.001). Discrepancies were often due to rare cases lacking guideline information or misunderstandings of case presentations.

**Conclusion:**

GPT-4 exhibited high compatibility with MTB decisions in cancer patient management, suggesting its potential as a supportive tool in clinical oncology. However, limitations exist, especially in rare or complex cases.

**Supplementary Information:**

The online version contains supplementary material available at 10.1007/s12094-025-03905-1.

## Introduction

Artificial intelligence (AI) is increasingly becoming a vital part of healthcare, offering new ways to support clinical decision-making. In oncology, treating cancer patients is a complex, multidisciplinary process that often requires input and suggestions from various specialties. Multidisciplinary tumor boards (MTBs) play a crucial role by bringing together experts from different fields to develop comprehensive treatment and diagnostic plans tailored to individual patients [1]. While MTBs enhance patient care by integrating diverse expertise, they can be resource-intensive and time-consuming, and they are not available in every oncology center worldwide.

Recent advancements in AI, particularly large language models (LLMs) like OpenAI’s GPT-4, have shown remarkable abilities in understanding and generating human-like responses. These models can interpret clinical data, extracting imaging features, summarize patient information, and suggest potential treatment options based on established guidelines [2, 3]. Also the recent literature showed a potential for personalized precision oncology with some limitations [4]. GPT-4 is shown to have improvements in breast cancer screening when compared with the previous model GPT-3.5 [5]. Fine-tuned LLMs are shown to have a potential for target volume contouring in radiotherapy, further enhancing the potential of AI in cancer care when compared to unimodal AI [6]. LLMs also showed outstanding performance in advance care planning from self-extracted data with electronic health records in advanced cancer patients [7]. This progress opens up the possibility of using AI to assist in clinical decision-making in oncology, potentially alleviating the workload of MTBs.

However, the extent to which AI models like GPT-4 can replicate the complex decision-making processes of MTBs remains unclear. Previous research has explored AI applications in oncology for tasks such as decision-making in several tumor sites, predicting cancer outcomes and identifying treatment targets [8–11]. Yet, there is limited knowledge about the ability of AI language models to align with multidisciplinary clinical decisions in real-world settings.

The primary objective of this study is to assess the consistency between MTB decisions and GPT-4’s treatment recommendations for cancer patients and to determine the causes of any inappropriate recommendations. By analyzing real-world data from patients discussed at MTBs in our center, we aim to evaluate how well GPT-4’s suggestions match those of experienced clinicians across various cancer types. This could help identify the potential role of AI models as supportive tools in clinical oncology, assisting in treatment planning and decision-making, and provide insights for further developments of AI in clinical decision-making.

## Methods

### Study design

This retrospective cross-sectional study was conducted at Ankara University Hospitals, a major reference center in Türkiye. Patients presented at the multidisciplinary tumor board between February 2021 and June 2023 were gathered from board registry notes and assessed for eligibility. The study did not involve any site-specific tumor boards or patients. The primary outcome was to assess the consistency between the multidisciplinary tumor board results and GPT-4 model predictions. Secondary outcomes included determining the appropriateness of incompatible results and the causes of any discrepancies.

### Multidisciplinary tumor board structure

The multidisciplinary tumor board at Ankara University Hospitals (Ankara, Türkiye) convenes weekly, both in-person and online, organized by the Medical Oncology Department. Invitation letters are sent to corresponding physicians via e-mail, including a list of patients to be discussed. Invited specialties include radiation oncology, general surgery, surgical oncology, gynecology and obstetrics, gynecological oncology, pathology, radiology, nuclear medicine, orthopedics, thoracic surgery, chest diseases, and ear–nose–throat diseases. When opinions from other specialties are necessary, they are also invited by e-mail.

This multidisciplinary tumor board differs from the site-specific tumor boards at Ankara University Hospitals, such as the breast cancer board, colorectal cancer board, thoracic surgery board, and hepatobiliary neoplasms board.

Additionally, a summary of the Turkish healthcare system and its medical specialties related to management of cancer patients is provided in the supplementary appendix to offer context regarding the clinical environment and specialty structures relevant to this study. Understanding the specific organization and roles within the Turkish healthcare system is essential for interpreting the study’s findings and assessing their applicability to other healthcare settings.

### Participants

All patients aged ≥ 18 years with a definite or prominently suspicious cancer diagnosis, regardless of site, were screened from the board registry notes. All such patients were included in the study. Exclusion criteria were lack of information about disease course, benign histological diagnosis, patients younger than 18 years, and absence of board results in registries. For patients presented in consecutive boards, only the last board presentation was included.

### Data collection

Collected data included the multidisciplinary tumor board date, attended specialties, number of cases presented, presenting specialties, patient age, cancer diagnosis, comorbidities, purpose of presentation and discussion, summary of disease course and treatments, and board results. This information was stored in a dedicated encrypted database, and any personal identifiers were censored during data collection.

The language model used for board result prediction was GPT-4 in ChatGPT (OpenAI Inc, California, USA). While this study was conducting, newer version of GPT model (GPT-4o) made publicly available, but as there is a risk of misinterpretation of results, the study was only conducted with GPT-4 model. After a brief introductory prompt regarding the purpose of the study and desired responses, cases were presented in the format: "…-year-old male/female patient with a diagnosis of …, disease and treatment course …, presentation purpose …". The cases were pre-processed to exclude any identity-informing data while protecting the essential information like comorbidities and performance status that is argued in the boards. Results were collected into raw text files. If any flaws were detected in the case understanding upon inspection of the generated response or any signs of hallucination, corrections were made, the case was re-submitted with the amendments, and any prompt modifications or prompt-engineering methods were avoided to lower the risk of attenuation bias. When significant changes in treatment suggestions occurred over time, the prompt included a note that the response should reflect the relevant approximate date. If a suggested treatment was not available or reimbursed in Türkiye, the correction prompt included "the … treatment is not available." Example prompts for different cases are available in the supplementary appendix. After response collection, the results were encoded in the database for further analysis.

### Analyses of results and statistical analysis

After collecting the responses, the board results and GPT-4 model predictions were grouped into several categories to enhance conciseness of the results, and three raters blindly evaluated the compatibility, defined as the consistency of discussion on the tumor board and GPT-4 model’s predictions in the study protocol. Compatibility was assessed on a scale ranging from 1 to 4, where 1 indicated total incompatibility, 3 indicated some incompatibilities but still appropriate, and 4 indicated major compatibility. All the blinded raters have agreed on the compatibility scores in sample fictional cases to avoid misinterpretations. The compatibility scores above 2 were accepted as appropriate, and a compatibility threshold was set at 2 for further assessment of suitability. Incompatible results were reviewed by two expert oncologists for appropriateness, and details of any inappropriateness were documented. The details and interpretation of the proposed scale are available in the supplementary appendix.

All statistical analyses were performed using R version 4.4.1 (R Foundation for Statistical Computing, Vienna, Austria). Descriptive statistics for categorical variables, such as tumor sites and presenting specialties, were presented as counts and percentages. Non-normally distributed continuous variables were shown as medians and interquartile ranges (IQR), while normally distributed continuous variables were presented as means and standard deviations (SD). Interrater consistency was calculated using Cronbach’s α test, and the assessment of suitability by expert oncologists was calculated using Cohen’s κ test. One-way analysis of variance (ANOVA) test was implemented to assess the mean differences in different disease status and post hoc tests were done with Games–Howell test. The temporal change in mean rater scores were assessed in six time points over time to address subtle updates or self-learning of the model with previous prompts, and the results were explained with time-series analysis.

### Ethical statements

Institutional ethics committee approved the study (Ankara University, Faculty of Medicine, Ankara, Türkiye, Approval Number: AUTF-KAEK 2024/358, Approval Date: 25.6.2024). The consent to participate was waived from ethics committee due to retrospective nature.

## Results

### Details of multidisciplinary tumor boards and patients presented

**Fig. 1:**
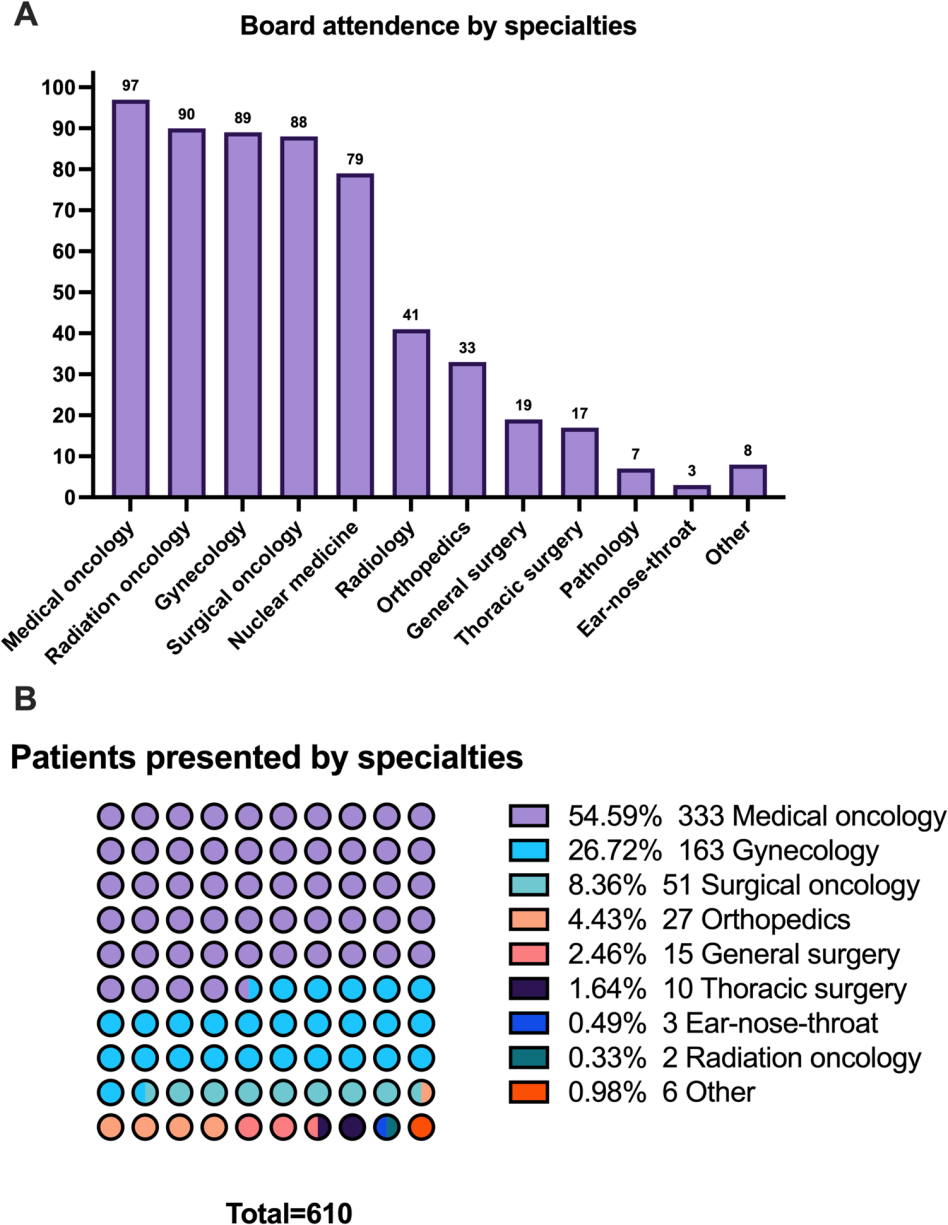
Board attendance and patients presented by specialties. A: Board attendance by specialties; B: patients presented by specialties

Ninety-seven MTBs held between February 2021 and June 2023 were included in the study. Medical oncology attended all boards, followed by radiation oncology at 90 boards (92.8%), gynecology and obstetrics at 89 boards (91.8%), surgical oncology at 88 boards (90.7%), nuclear medicine at 79 boards (81.4%), and radiology at 41 boards (42.3%). A total of 610 patients were presented and included in the study. Most patients were presented by medical oncology with 333 patients (54.6%), followed by gynecology and obstetrics with 163 patients (26.7%) and surgical oncology with 51 patients (8.4%). The median number of patients presented per board was 6 (IQR 4–8). Details of the multidisciplinary tumor boards and patients presented by specialties are shown in Table 1 and Fig. 1.

**Table 1:** Board attendance and patients presented by specialties

Specialties	Number of boards attended,* n* (%)	Patients presented, * n* (%)
Medical oncology	97 (100%)	333 (54.6%)
Radiation oncology	90 (92.8%)	2 (0.3%)
Gynecology and obstetrics	89 (91.8%)	163 (26.7%)
Surgical oncology	88 (90.7%)	51 (8.4%)
Nuclear medicine	79 (81.4%)	0
Radiology	41 (43.2%)	0
Orthopedics	33 (34.0%)	27 (4.4%)
General surgery	19 (19.6%)	15 (2.5%)
Thoracic surgery	17 (17.5%)	10 (1.6%)
Pathology	7 (7.2%)	0
Ear-nose-throat	3 (3.1%)	3 (0.5%)
Other*	8 (8.2%)	6 (1.0%)

*Other specialties include cardiology, endocrinology, dermatology, and hematology

**Fig. 2:**
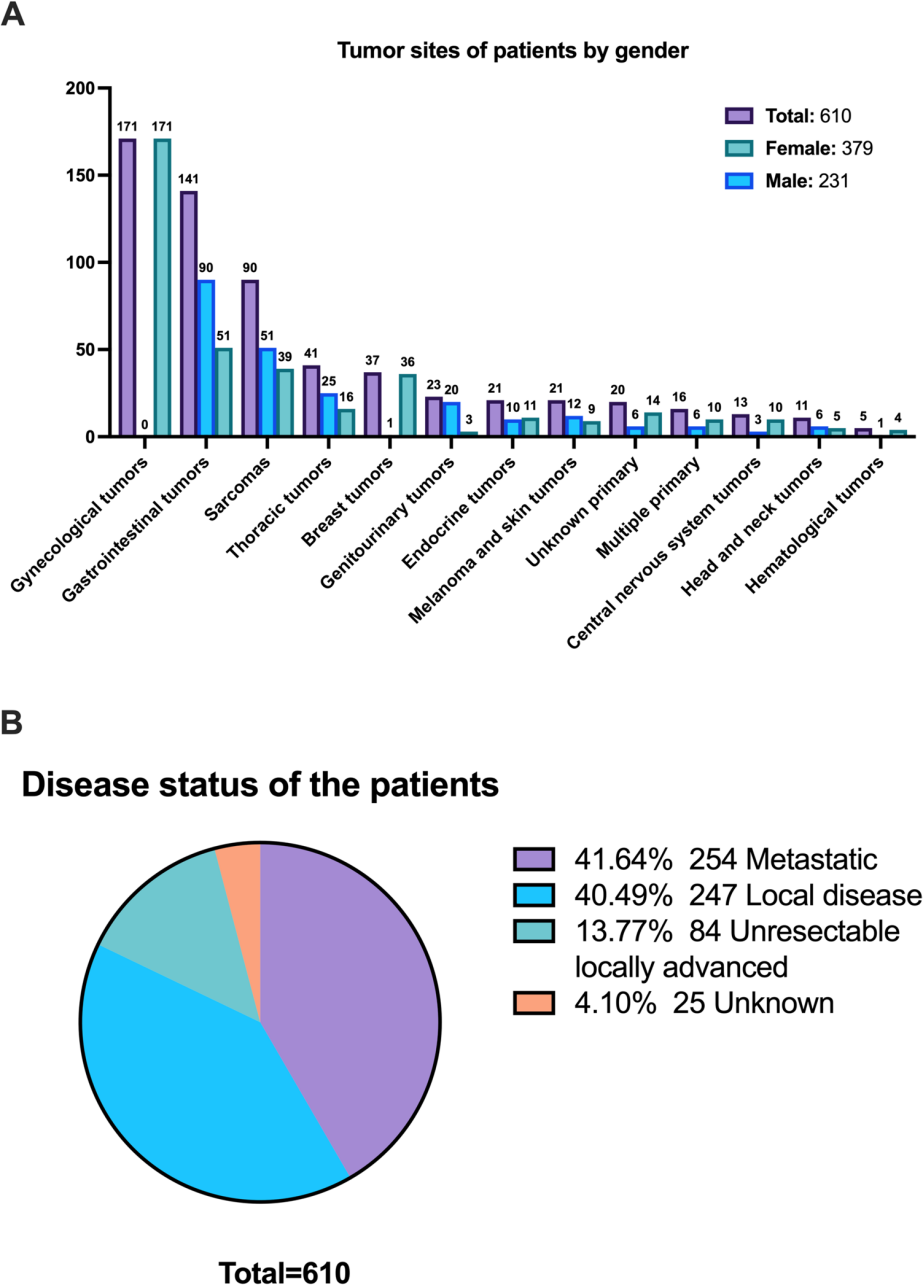
Primary tumor sites by genders and disease status of the patients.** A** Primary tumor sites by gender.** B** Disease status of the patients

The median age of patients was 57 years (IQR 43–66); 379 patients (62.1%) were female, and 231 patients (37.9%) were male. The median age was 58 years (IQR 47–65) for female patients and 54 years (IQR 40–66) for male patients. Female patients were significantly older than male patients (Mann–Whitney *U* test, *p* = 0.047). The most common primary diagnoses were gynecological tumors (171 patients, 28.0%), followed by gastrointestinal tumors (141 patients, 23.1%) and sarcomas (90 patients, 14.8%). The main purpose of presentation was to determine the treatment plan (583 presentations, 95.7%), followed by surveillance planning (13 presentations, 2.1%) and diagnostic approach (9 presentations, 1.5%). 254 patients (41.6%) had metastatic disease, 247 patients (40.5%) had local disease, 84 patients (13.8%) had unresectable locally advanced disease, and 25 patients (4.1%) had unknown disease status. The tumor sites of presented patients by gender and disease status of the patients are shown in Fig. 2. Further details and patients’ comorbidities prompted in analyses are provided in the supplementary appendix.

### Compatibility of multidisciplinary tumor board results and predictions of GPT-4 model

**Fig. 3:**
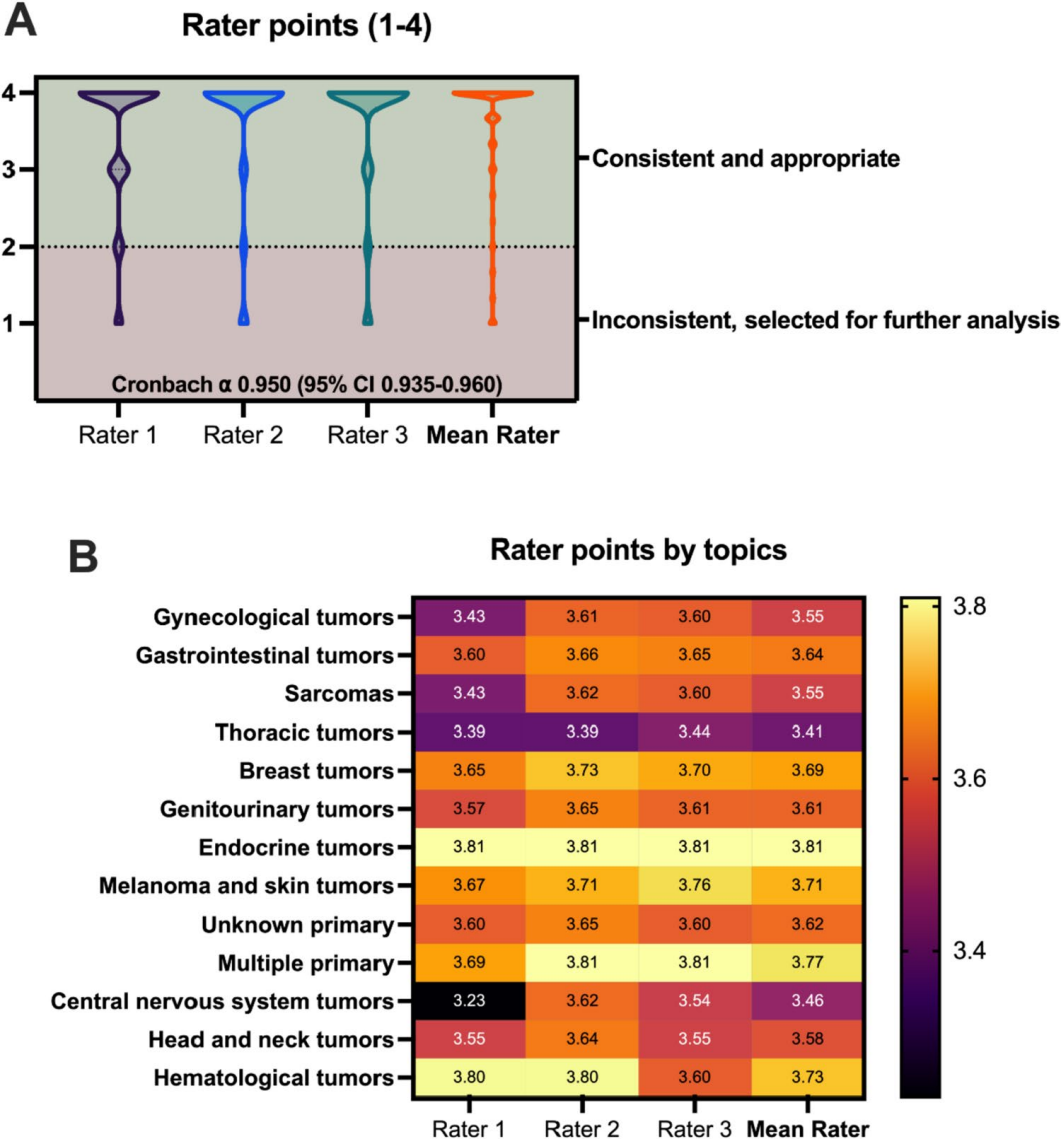
Rating of observers and ratings by topics. A: Rater points in violin plot, dashed line is the threshold of 5 for further analysis; B: heatmap of mean rater points by topics

The mean compatibility score across all three raters was 3.59 (SD = 0.81), with individual scores of 3.52 (SD = 0.85), 3.64 (SD = 0.87), and 3.62 (SD = 0.85). Interrater reliability assessed with Cronbach’s α test yielded an alpha value of 0.950 (95% CI 0.935–0.960), indicating high reliability and suitability for making important decisions. Mean compatibility scores by topic ranged from 3.41 in thoracic tumors to 3.81 in endocrine tumors. The lowest compatibility score was 3.23 in central nervous system tumors, and the highest was 3.81 in endocrine tumors. Major compatibility (4 points on the scale) was observed in 412 cases (67.5%), while 16 cases (2.6%) were totally incompatible. Graphical comparisons and details of the rater points are presented in Table 2 and Fig. 3.

**Table 2:** Rater points by tumor sites

Tumor sites	Rater 1 points (SD)	Rater 2 points (SD)	Rater 3 points (SD)	Mean points (SD)
Gynecological tumors	3.43 (SD = 0.9)	3.61 (SD = 0.94)	3.6 (SD = 0.92)	3.55 (SD = 0.87)
Gastrointestinal tumors	3.6 (SD = 0.81)	3.66 (SD = 0.84)	3.65 (SD = 0.81)	3.64 (SD = 0.78)
Sarcomas	3.43 (SD = 0.89)	3.62 (SD = 0.83)	3.6 (SD = 0.79)	3.55 (SD = 0.79)
Thoracic tumors	3.39 (SD = 0.97)	3.39 (SD = 1.05)	3.44 (SD = 1.03)	3.41 (SD = 0.99)
Breast tumors	3.65 (SD = 0.72)	3.73 (SD = 0.65)	3.7 (SD = 0.7)	3.69 (SD = 0.66)
Genitourinary tumors	3.57 (SD = 0.84)	3.65 (SD = 0.93)	3.61 (SD = 0.94)	3.61 (SD = 0.89)
Endocrine tumors	3.81 (SD = 0.51)	3.81 (SD = 0.6)	3.81 (SD = 0.51)	3.81 (SD = 0.49)
Melanoma and skin tumors	3.67 (SD = 0.8)	3.71 (SD = 0.9)	3.76 (SD = 0.77)	3.71 (SD = 0.8)
Unknown primary	3.6 (SD = 0.82)	3.65 (SD = 0.93)	3.6 (SD = 0.88)	3.62 (SD = 0.83)
Multiple primary	3.69 (SD = 0.77)	3.81 (SD = 0.8)	3.81 (SD = 0.71)	3.77 (SD = 0.73)
Central nervous system tumors	3.23 (SD = 1.01)	3.62 (SD = 0.87)	3.54 (SD = 0.97)	3.46 (SD = 0.9)
Head and neck tumors	3.55 (SD = 0.69)	3.64 (SD = 0.92)	3.55 (SD = 0.93)	3.58 (SD = 0.82)
Hematological tumors	3.8 (SD = 0.45)	3.8 (SD = 0.45)	3.6 (SD = 0.89)	3.73 (SD = 0.59)
Total	3.52 (SD = 0.85)	3.64 (SD = 0.87)	3.62 (SD = 0.85)	3.59 (SD = 0.81)

The mean compatibility scores for patients with metastatic disease were 3.61 (SD = 0.78), local disease were 3.52 (SD = 0.90), unresectable locally advanced were 3.65 (SD = 0.72), and unknown disease status was 3.99 (SD = 0.06). One-way ANOVA test showed significance (*F* = 41.6, *p* < 0.001) and post hoc tests showed that compatibility scores of unknown disease status were all significant when compared to other groups (*p* < 0.001).

In time-series analysis, the mean compatibility scores ranged from 3.45 (SD = 0.96) in fifth time point to 3.75 (SD = 0.61) in second time point. The change over time showed increasing and decreasing mean rater scores with a slight tendency of increasing over time with minimal changes in standard deviation. The graphical presentation of time-series analysis is presented in supplementary appendix.

### Appropriateness of the results below the suggested threshold

Sixty-two patients (10.2%) had a mean compatibility score below the suggested threshold of 2 points. The first expert oncologist determined that the GPT-4 model's prediction was inappropriate in 8 of these 62 patients (12.9%), while the second expert oncologist found it inappropriate in 16 of 62 patients (25.8%). Cohen’s *κ* analysis showed moderate agreement between the experts, with a κ value of 0.50 (95% CI 0.25–0.75), which was statistically significant (*p* < 0.001).

The first expert oncologist noted that all 8 inappropriate predictions were due to a lack of information online and in guidelines because of the rarity of the cases. The second expert oncologist also attributed 8 inappropriate predictions to a lack of information and an additional 8 to misunderstandings of the case presentation. Disagreements were observed in 10 cases where eligibility was assessed by both oncologists. Summaries of the appropriateness of the results and expert opinions are presented in Table 3.

**Table 3:** Appropriateness of the predicted results and agreement among expert opinions

Appropriateness	Expert oncologist 1, *n* (%)	Expert oncologist 2, *n* (%)
Appropriate	54 (87.1%)	46 (74.2%)
Inappropriate	8 (12.9%)	16 (25.8%)

Disagreements	Expert oncologist 1 appropriate	Expert oncologist 2 appropriate, n (%)
Expert oncologist 1 inappropriate	–	1/62
Expert oncologist 2 inappropriate	9/62	–

## Discussion

In this cross-sectional study involving 610 cancer patients presented at multidisciplinary tumor boards (MTBs), we assessed the compatibility of GPT-4’s treatment recommendations with the decisions made by MTBs. The findings revealed a high level of agreement between GPT-4 predictions and MTB decisions, with a mean compatibility score of 3.59 out of 4 across all cases. The interrater reliability among the three independent raters was excellent, demonstrated by a Cronbach’s alpha of 0.950, indicating consistent assessments of compatibility. These results imply that GPT-4 generated suggestions are relevant to clinical professionals and shows outstanding potential for decision-making in oncology practice.

The majority of GPT-4’s recommendations aligned closely with the MTB decisions, suggesting that the AI model effectively captured the standard treatment approaches utilized in clinical oncology. Major compatibility (a score of 4) was achieved in 67.5% of the cases, highlighting the model’s capability to generate treatment suggestions that are entirely consistent with expert clinical judgments. The compatibility scores of patients with unknown disease status were near the total compatibility score of 4, significantly higher than the other groups. When exploring the cause, MTB and GPT-4 suggestions were mostly performing biopsy and imaging with same modalities. This result can be interpreted as AI LLMs potential for diagnostic assistance.

Discrepancies were observed in 10.2% of the cases, primarily involving rare or complex cancer types. Upon further evaluation by expert oncologists, GPT-4’s recommendations were deemed inappropriate in a minority of these cases—12.9% by the first oncologist and 25.8% by the second—indicating that while the AI model performs well overall, it has limitations in certain clinical scenarios like rare tumors and lack of information in guidelines. The moderate agreement between the expert oncologists (Cohen’s *κ* = 0.50) reflects the inherent challenges and subjective nuances in clinical decision-making for complex cases. The lack of information in the training sets, limited knowledge in internet and literature, and complex nature of the cancer care may be the major cause of this results, presenting the main limitation of GPT-4 of in rare and complex cancer cases.

Several studies have explored the performance of LLMs like GPT-3.5 and GPT-4 in oncology settings. In a study by Sorin et al. suggested that when the GPT-3.5 model’s responses graded between 1 and 5, the grades of two reviewers ranged between 3.7 and 4.6, and the results are suggestive of using LLMs as a decision support tool [10]. In contrast, Lukac et al. suggested that GPT 3.5 lacked the ability to provide specific recommendations about breast cancer patients [9]. Benary et al. explored the ability of four LLMs in precision oncology in 4 LLMs including GPT-4, and showed the limitations of models, but as this study conducted before the reasoning algorithms, the insufficiency of AI systems in precision oncology may be deprecated [4]. Griewing et al. observed a 58.8% concordance between MTBs and GPT-3.5, limiting its usability. However, these studies relied on earlier versions of GPT-3.5, and the results should be interpreted accordingly. Liang et al. emphasized that GPT-4 outperformed GPT-3.5 in renal oncology, suggesting that recent developments enhance the reliability of LLMs [12]. Lechien et al. suggested that GPT-4 model was also provided reliable recommendations about TNM and biopsy indications but had limitations in decision-making in head and neck cancers [11]. Although not focusing on multidisciplinary settings, Kozel et al. reported that GPT-4 achieved 85% accuracy in brain tumor diagnosis and 75% in treatment planning [13]. Rao et al. also showed the improvement in breast cancer radiological decision-making when comparing GPT-4 to GPT 3.5 [5]. Our study had better precision that may be attributed to the temporal evolution of LLMs, as most of the literature focused on GPT-3.5, the previous version of GPT-4 which we used in our study, and these studies are confirming our hypothesis of evolving nature of AI in clinical practice.

Luo et al. showed that commercially available LLMs struggle to extract patient-centered outcomes without fine-tuning, which aligns with our findings that GPT-4 struggled in providing correct results for rare patients and in the absence of guidelines [14]. Other studies found that providing detailed prompts improved the diagnostic performance of GPT-4 in pathological analysis and ophthalmological cases, highlighting the need for further training for LLMs [15, 16]. Conversely, Huang et al. reported that GPT-3.5 demonstrated high performance in clinical data extraction from pathology notes [17].

Developing AI for clinical applications is a challenging and time-consuming process that requires a multidisciplinary approach. [18]This is exemplified by the Neuro-GPT-X project by Guo et al.; despite being trained on over 4,000 articles on schwannomas, it does not demonstrate superior performance compared to the general-purpose GPT-4 model [19]. Also, CancerGPT model developed by Li et al. with approximately 124 million parameters showed drug pair synergy prediction potential of LLMs, further implying the potential of LLMs in personalized cancer care that can be utilized in limited resources [18]. Another example is a cancer patient companion chatbot developed by Lee et al., which utilized an integrated meta-dataset of 1.17 million tokens and involved an extensive development process [20].

GPT-4 has been shown to have high sensitivity but low specificity in the histopathological classification of colorectal adenomas, highlighting a major limitation of AI in cancer care [21]. Aligning with this study, Kaiser et al. proposed that LLMs have limited ability to determine the later line treatments in colorectal cancer cases, discussing the limitations and need for further developments in AI before implementing in clinical decision process [22]. Moreover, the multidisciplinary approach integrating histology, pathology, and oncology is crucial for accurate diagnosis and effective treatment planning, as it leverages the collective expertise of various specialists to address complex cancer cases [23].

The results of this study highlight the transformative potential of artificial intelligence (AI) models like GPT-4 in the field of oncology. In our other research of Turkish Society of Medical Oncology’s annual board examinations, the LLM’s showed potency to answer questions, above passing the suggested threshold, but had a limitation in recent developments [24]. The high compatibility between GPT-4 predictions and multidisciplinary tumor board (MTB) decisions demonstrates that AI can effectively support clinical decision-making processes. By providing evidence-based treatment recommendations, AI can help streamline the decision-making workflow, reduce the cognitive burden on healthcare professionals, and ensure consistency with the latest clinical guidelines. This integration of AI into clinical practice has the potential to enhance the quality of patient care, improve treatment outcomes, and optimize the allocation of healthcare resources.

The study also underscores the importance of a collaborative approach between AI developers and healthcare professionals to address the current limitations of AI models. Specifically, the challenges observed in rare or complex cancer cases indicate that AI systems must be continuously refined and tailored to handle diverse clinical scenarios. Developing dedicated AI systems that are customized to specific clinical environments and continuously updated with the latest medical knowledge is crucial for enhancing their reliability and applicability. Additionally, fostering ongoing education and training for clinicians on the effective use of AI tools is essential to maximize their benefits and ensure seamless integration into existing clinical workflows. Future advancements should focus on creating robust, adaptable AI frameworks that complement the expertise of multidisciplinary teams, thereby fostering a synergistic relationship where AI-driven insights enhance human decision-making. This collaborative paradigm not only improves the accuracy and efficiency of clinical decisions but also builds trust in AI technologies, facilitating their broader adoption in oncology and other medical specialties.

### Strengths and limitations

This study's primary strength lies in its pioneering assessment of GPT-4's compatibility with multidisciplinary tumor board (MTB) decisions using real-world data from a substantial in a large sample of 610 patients. The inclusion of diverse cancer types enhances the generalizability of the findings across various oncological scenarios. The methodology is robust, featuring three independent raters and demonstrating excellent interrater reliability (Cronbach’s α = 0.950), which adds credibility to the compatibility assessments between GPT-4 predictions and MTB decisions. Also, the time-series analysis contributes to reliability of this study as no subtle updates or self-learning capability was shown.

However, the study has certain limitations. Being a single-center, retrospective study conducted in Türkiye, the results may not be fully generalizable to other healthcare settings with different clinical practices and patient demographics. While some measures taken, there is a risk of attention bias due pre-processing of the prompts. Additionally, the GPT-4 model showed limitations in handling rare or complex cancer cases, often due to a lack of comprehensive guideline information or misunderstandings from case presentations. Also, lacking newer machine learning algorithms like reasoning may affect this study’s generalizability. These factors highlight the need for caution when applying AI models in less common clinical scenarios and underscore the necessity for further refinement and validation in diverse settings.

## Conclusion

This single-center retrospective study demonstrated that the GPT-4 language model exhibits a high level of compatibility with multidisciplinary tumor board (MTB) decisions in cancer patient management. With a mean compatibility score of 3.59 out of 4 and excellent interrater reliability (Cronbach’s *α* = 0.950), GPT-4 shows significant promise as a supportive tool in clinical oncology. The model effectively mirrored MTB recommendations in the majority of cases, suggesting its potential utility in aiding treatment planning and decision-making processes.

However, the study also revealed limitations, particularly in rare or complex cancer cases where discrepancies were more prevalent. These inconsistencies were often attributed to a lack of guideline information available to the AI model or misunderstandings arising from case presentations. This underscores the current challenges faced by AI models like GPT-4 in handling less common clinical scenarios and highlights the necessity for further refinement.

In conclusion, while GPT-4 demonstrates substantial potential to complement clinical decision-making in oncology, especially for more common cancer types, there is a clear need for ongoing development. Enhancing the model's ability to process complex and rare cases by incorporating more comprehensive and up-to-date medical guidelines could improve its applicability. Future research should focus on refining AI models to ensure that they can provide accurate and reliable recommendations across the full spectrum of oncological cases, ultimately supporting healthcare professionals in delivering optimal patient care.

## Supplementary Information

Below is the link to the electronic supplementary material.

Supplementary file1 (DOCX 101 KB)

## Data Availability

Due to strict regulations in Türkiye, the data cannot be made publicly available. Extracted and censored data will be shared upon reasonable request.
