# Supplementary material for: Assessing the accuracy of the GPT-4 model in multidisciplinary tumor board decision prediction
Source: Clin Transl Oncol. 2025 Mar 25;27(9):3793–802. doi: 10.1007/s12094-025-03905-1 (PMC12399707; doi:10.1007/s12094-025-03905-1)
Supplement: Supplementary file 1 — Supplementary file1 (DOCX 101 KB) [file 12094_2025_3905_MOESM1_ESM.docx]

Supplementary Appendix

[1. Brief overview of Turkish healthcare system and oncology related specialties 2](#_Toc192062320)

[2. Simulated sample prompts 3](#_Toc192062321)

[3. Compatibility scale details 4](#_Toc192062322)

[4. Diagnosis of patients by gender 5](#_Toc192062323)

[5. Temporal change in mean rater scores 7](#_Toc192062324)

[6. Significant comorbidities of the patients 8](#_Toc192062325)

# Brief overview of Turkish healthcare system and oncology related specialties

As the Turkish physician specialties and subspecialties differed from the other countries, we thought that it would be appropriate to provide a summary to give an insight of Turkish healthcare system. There isn't a clinical oncology specialty in Turkiye, and three major oncological specialties are present. The medical oncology is a subspecialty of internal medicine and primarily interested in medical treatment and follow-up of cancer patients, and hematology is separated from medical oncology in Turkish healthcare system. Radiation oncology is primarily interested in radiotherapy and follow-up of patients received radiotherapy. Surgical oncology is a subspecialty of general surgery primarily interested in surgical management of cancer patients of all sites except brain tumors. All the surgical specialties are permitted to do surgical managements of cancer patients in relevant sites, but radiotherapy permission is limited to radiation oncology and except medical oncology, the specialties have limited systemic treatment permission.

# Simulated sample prompts

- 50 years old male patient with rectal carcinoma, colonoscopy showed fragile mass within 10 cm of anal verge. The biopsy showed adenocarcinoma, pelvic MRI showed at least T3N1 disease.
- 35 years old female, operated for medulloblastoma and received craniospinal radiotherapy, adjuvant chemotherapy?
  - Lomustine is not available in Turkey
- 60 years old male patient, dMMR metastatic colorn cancer, BRAF and RAS wild-type, received FOLFOX + bevacizumab, FOLFIRI + cetuximab, regorafenib, TAS-102 + Bevacizumab, CAPOX. Only liver metastatic in CT and MRI
  - Liver metastases are not feasible for local ablative treatment, suggest according to September 2019
- 32 years old female patient with Rb gene mutation, operated for bilateral retinoblastoma while infancy, osteosarcoma of left thigh operated 2 years ago, CT showed new lesion on lung, further evaluation plan
- 54 years old female patient, FIGO stage 2B cervix cancer. Chronic kidney disease, eGFR is 54 ml/min, adjuvant treatment
- 80 years old female patient with metastatic squamous cell cancer of unknown primary with PET-CT showed metastasis on liver
  - Cemiplimab is not available

# Compatibility scale details

| Scale points | Evaluation |
| --- | --- |
| 4 | Almost exactly the same, major compability |
| 3 | Same with some differences, appropriate |
| 2 | Different with some consistencies |
| 1 | Almost exactly different |

# Diagnosis of patients by gender

|  | **Male** | **Female** | **Total** |
| --- | --- | --- | --- |
| **Ovarian cancer** | 0 | 69 | **69** |
| **Endometrial cancer** | 0 | 55 | **55** |
| **Colon cancer** | 33 | 8 | **41** |
| **Breast cancer** | 1 | 36 | **37** |
| **NSCLC** | 19 | 9 | **28** |
| **Rectum cancer** | 13 | 11 | **24** |
| **Undifferantiated/pleomorphic sarcoma** | 14 | 10 | **24** |
| **Gastric cancer** | 15 | 6 | **21** |
| **Unknown primary** | 7 | 14 | **21** |
| **Carcinoid tumor/neuroendocrine tumor** | 8 | 11 | **19** |
| **Cervical cancer** | 0 | 17 | **17** |
| **Multiple primary** | 8 | 9 | **17** |
| **Osteosarcoma** | 9 | 8 | **17** |
| **Ewing sarcoma** | 7 | 7 | **14** |
| **Leiomyosarcoma** | 1 | 13 | **14** |
| **Malign melanoma** | 6 | 6 | **12** |
| **Hepatocellular cancer** | 8 | 3 | **11** |
| **Testicular cancer** | 10 | 0 | **10** |
| **Uterine carcinosarcoma** | 0 | 9 | **9** |
| **Basal cell carcinoma** | 5 | 3 | **8** |
| **Desmoid tumor** | 5 | 3 | **8** |
| **Glial tumor/glioblastoma multiforme** | 1 | 7 | **8** |
| **Mullerian adenocarcinoma** | 0 | 8 | **8** |
| **Appendix cancer** | 1 | 6 | **7** |
| **Adrenocortical cancer** | 4 | 2 | **6** |
| **Cholangiocarcinoma** | 5 | 1 | **6** |
| **Chondrosarcoma** | 3 | 3 | **6** |
| **Gastrointestinal stromal tumor** | 2 | 4 | **6** |
| **Thyroid cancer** | 2 | 4 | **6** |
| **Liposarcoma** | 4 | 1 | **5** |
| **Synovial sarcoma** | 3 | 2 | **5** |
| **Thymoma/thymic cancer** | 4 | 1 | **5** |
| **Lymphoma** | 1 | 3 | **4** |
| **Pancreatic cancer** | 3 | 1 | **4** |
| **Pheochromocytoma/paraganglioma** | 3 | 1 | **4** |
| **Renal cell carcinoma** | 4 | 0 | **4** |
| **SCLC** | 1 | 3 | **4** |
| **Bladder cancer** | 1 | 2 | **3** |
| **Esophageal cancer** | 0 | 3 | **3** |
| **Larynx cancer** | 3 | 0 | **3** |
| **Prostate cancer** | 3 | 0 | **3** |
| **Tongue cancer** | 2 | 1 | **3** |
| **Adenoid cystic carcinoma** | 0 | 2 | **2** |
| **Anal cancer** | 1 | 1 | **2** |
| **Angiosarcoma** | 0 | 2 | **2** |
| **Fallopian tube tumor** | 0 | 2 | **2** |
| **Medulloblastoma** | 0 | 2 | **2** |
| **Mesothelioma** | 0 | 2 | **2** |
| **Primary peritoneal cancer** | 0 | 2 | **2** |
| **Rhabdomyosarcoma** | 2 | 0 | **2** |
| **Wilms tumor** | 1 | 1 | **2** |
| **Chordoma** | 1 | 0 | **1** |
| **Ependymoma** | 0 | 1 | **1** |
| **Giant tumor of bone** | 1 | 0 | **1** |
| **Histiocytosis** | 1 | 0 | **1** |
| **Hypopharynx cancer** | 0 | 1 | **1** |
| **Malignant schwannoma** | 1 | 0 | **1** |
| **Myoepithelial tumor of skin** | 1 | 0 | **1** |
| **Neurofibromatosis** | 1 | 0 | **1** |
| **Partial mole** | 0 | 1 | **1** |
| **Sinonasal carcinoma** | 0 | 1 | **1** |
| **Small bowel cancer** | 1 | 0 | **1** |
| **Vaginal cancer** | 0 | 1 | **1** |
| **Periampullary tumor** | 1 | 0 | **1** |

# Temporal change in mean rater scores

# Significant comorbidities of the patients

| **Low performance status (ECOG PS 4)** | 11 |
| --- | --- |
| **Chronic kidney disease** | 7 |
| **Cirrhosis** | 7 |
| **Hyperbilirubinemia** | 5 |
| **Morbid obesity** | 5 |
| **Cerebrovascular accident** | 4 |
| **Congestive heart failure** | 4 |
| **Coronary arterial disease** | 3 |
| **Chronic obstructive pulmonary disease** | 3 |
| **Bone marrow involvement** | 2 |
| **Paraplegia** | 2 |
| **Pulmonary embolism** | 2 |
| **Liver transplantation** | 1 |
| **Fulminant ulcerative colitis** | 1 |
| **Essential thrombocytosis** | 1 |
